# Case series analysis of eight underground tunnellers with chronic silicosis in Queensland

**DOI:** 10.1002/rcr2.756

**Published:** 2021-05-07

**Authors:** Kalesh Seevnarain, Nicholas Burke, Katrina Newbigin

**Affiliations:** ^1^ 4cRisk Pty Ltd Brisbane Queensland Australia; ^2^ I Med Radiology Brisbane Queensland Australia

**Keywords:** ILO chest radiograph, occupational lung disease, pneumoconiosis, silicosis, tunnellers

## Abstract

Within Australia, chronic silicosis has been long thought of as being a well‐controlled occupational lung disease. While recent cases of acute silicosis in artificial stone benchtop cutters have emerged, chronic silicosis within the general workforce population has not been recorded. Our case series describes the re‐emergence of chronic silicosis amongst workers within the tunnelling industry representing the potential for a more widespread insidious occupational lung disease. While undertaking pre‐employment medicals, eight tunnellers have been diagnosed with chronic silicosis. These eight tunnellers had a minimum of 10 years of cumulative dust exposure prior to diagnosis. Diagnosis was made by radiological evaluation of chest X‐rays and computerized tomography scans by International Labour Organization B Readers. The re‐emergence of chronic pneumoconiosis as illustrated by this case series suggests the presence of undiagnosed occupational lung disease with far reaching implications for workers, employers, compensation systems, and the public healthcare sectors.

## Graphical abstract

This article outlines the emergence of chronic silicosis in a population which has been previously thought as not being at risk for pneumoconiosis. It also outlines the concerns around fragmented reporting and compensation systems.

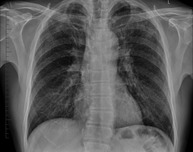

## Introduction

Silicosis is a well‐recognized interstitial lung disease which has been described as early as 430 BC by Hippocrates [1]. All types of silicosis were thought to have been well controlled in Australia with a reported incidence rate of less than 1 claim per million workers per year being identified for pneumoconiosis other than asbestosis between the period of 1 January 1996 and 31 December 2003 [2]. However, case reports, audits, and outbreak investigations during 2017 described 799 cases of acute and accelerated silicosis amongst people working with engineered stone [3, 4]. While this cohort generated international and local attention due to the devasting outcomes associated with this specific disease profile, it was largely believed that silicosis was limited to this small sector with the larger workforce still being protected from this work‐related pneumoconiosis.

Silicosis is a spectrum of disease that is dependent on the cumulative dust exposure over time. Acute silicosis is associated with significantly high dust levels over short periods of time with a poor prognosis and in most cases mortality within five years. Chronic silicosis has a longer latency period of greater than 10 years. While chronic silicosis is initially asymptomatic, as the disease progresses, a worker may develop chronic obstructive airways disease, congestive cardiac failure, and be at increased risk of other diseases such as tuberculosis and lung cancer [1].

In February 2020, new guidance around medical surveillance for workers exposed to respirable crystalline silica has been issued by Safe Work Australia [5, 6]. This called for the performance of chest radiography and evaluation by specialist radiologists familiar with the pneumoconiosis. In addition, this guidance suggested that computerized tomography could also be used in surveillance programmes should abnormalities be detected. There has also been a decrease in the national workplace exposure limit for all forms of crystalline silica from 0.1 to 0.05 mg/m^3^ (8‐h time‐weighted average) [6].

## Case Series

### Methods

An analysis of pre‐employment medical examinations for those workers within the tunnelling industry, between December 2019 and December 2020, was undertaken. These medicals included occupational and medical histories, spirometry, and chest radiographs read by the International Labour Organization (ILO) B readers. Initial review of the pre‐employment medical examination results was undertaken by occupational and environmental medicine physicians. Initial screening was first conducted by using ILO chest radiographs with findings confirmed with a computerized tomography scan. All identified cases were also referred to respiratory physicians for evaluation of the lung disease severity. Standardized lung function testing was performed.

### Results

During this period, the review of 284 pre‐employment medicals was undertaken for persons exposed to silica working underground in tunnelling environments. This initial review identified 23 workers with comments on chest radiograph. Further internal review identified 11 workers with confirmed chronic silicosis. While 11 workers were confirmed with chronic silicosis, eight workers provided informed consent. Another worker was identified as having a false‐positive result, that is, the worker was thought to have radiological features on the initial ILO B read but was confirmed as negative by subsequent review of the ILO chest X‐ray and further computerized tomography scanning. The false‐positive case has been excluded from this case series.

Our case series of the eight workers comprised of only males with an average age of 49 years (range 40–62 years). All individuals identified in the case series had a history of working on large infrastructure and construction projects across Victoria and New South Wales before travelling to Queensland to commence work on projects. While there were various job descriptions within the eight cases, all cases had been working underground within tunnels as they were being constructed. The average occupational cumulative dust exposure time was 21 years (range 10–44 years) with two workers having a mineral mining history of greater than five years in conjunction to their tunnelling experience. All workers reported undergoing pre‐employment medical surveillance conducted for other jobs over the last 10 years. Workers from New South Wales also reported undergoing periodic medical surveillance while employed on projects in New South Wales.

The worker with the longest history of exposure (44 years) had been formally diagnosed and participated in an ongoing medical surveillance through a state‐based compensation system programme. In 2019, he had a reported ILO read of 2/3 with progressive massive fibrosis and was considered as fit to continue working with no advice being provided about suitable workplace control strategies. One worker had a differential diagnosis of silicosis in 2014 but was never followed up with our review providing him with the first formal diagnosis. Another worker had an ILO chest X‐ray of 1/0 with s/s nodules noted in 2019. No further investigations were performed at the time with the tunneller having been subsequently confirmed as 1/2 p/q in 2020.

**Figure 1:**
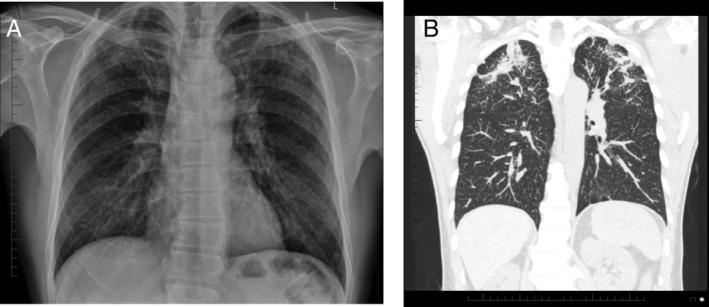
(A) A chest X‐ray reported with the International Labour Organization (ILO) standards in a tunneller with 30 years of dust exposure demonstrates silica nodules >3 mm in diameter with an ILO grade of 2/3. (B) Bilateral progressive massive fibrosis in the same tunneller was confirmed on a subsequent non‐contrast computerized tomography (CT) chest. This CT shows bilateral fibrotic masses with associated architectural distortion and volume loss on a background of diffuse solid silica nodules.

The remaining five workers were not aware of any diagnosis of silicosis being made prior to our review. The radiological features of a worker with 30 years of dust exposure has been illustrated in Figure 1 with a summary of the case series findings being descibed in Table 1.

**Table 1:** Summary of case series findings for eight tunnellers diagnosed with chronic silicosis during the period of December 2019 to December 2020.

	Age	Sex	Occupational history		Lung function test			Radiological findings		Diagnosis
			Job title	Dust exposure (years)	FEV_1_ L/min (%)	FVC L/min (%)	FEV_1_/FEV	ILO B reading	Pulmonary CT scan results	
Worker 1	44	Male	Operator (underground)	10	3.92 (90)	5.09 (92)	0.77	1/0 p/p	Multiple calcified nodules with bilateral lymphadenopathy. Emphysematous changes in upper and middle zones	Chronic silicosis with emphysema
Worker 2	40	Male	Tunneller	14	3.88 (89)	4.97 (92)	0.78	1/2 q/q	Numerous miliary nodules in both lungs with bilateral	Chronic silicosis
Worker 3	46	Male	Tunneller	14	4.05 (104)	4.84 (100)	0.83	1/0 q/s	Diffuse tiny pulmonary nodules	Chronic silicosis
Worker 4	48	Male	Line manager (tunnelling)	23	3.75 (87)	5.48 (101)	0.68	1/0 p/p	Multiple subcentimetre nodules in centrilobular distribution with calcification and hilar lymphadenopathy	Chronic silicosis
Worker 5	45	Male	Tunneller	11	3.25 (81)	4.33 (86)	0.75	1/0 p/p	Scattered pulmonary micronodules in both upper lobes	Chronic silicosis
Worker 6	58	Male	Tunneller	30	4.78 (115)	6.03 (111)	0.79	2/3 q/r	Bilateral large opacities, progressive massive fibrosis	Chronic silicosis with progressive massive fibrosis
Worker 7	62	Male	Line manager (tunnelling)	44	3.21 (92)	4.04 (90)	0.80	2/2 p/q	Right upper lobe large opacity, atelectasis, calcified lymphadenopathy	Chronic silicosis with progressive massive fibrosis
Worker 8	46	Male	Tunneller	23	3.06 (80)	5.32 (111)	0.72	1/2 p/q	Moderate diffuse mediastinal and bilateral lymphadenopathy with calcification. Nodules 1–6 mm. Moderate to severe centrilobular emphysema	Chronic silicosis with emphysema

CT, computerized tomography; FEV_1_, forced expiratory volume in 1 sec; FVC, forced vital capacity; ILO, International Labour Organization.

From a clinical perspective, all workers were asymptomatic for features of chronic lung disease. Two tunnellers had radiological evidence of emphysema with another tunneller also having features of lung atelectasis.

Attempts were made to source previous pre‐employment and periodic medical surveillance records for comparative analysis. However, as this information had not been directly provided to the workers and was gathered during employment for various other companies, attempts were unsuccessful. Information about personal protective equipment was also difficult to gather. Anecdotally, all workers especially those with longer exposure histories stated that workplace controls and personal equipment were limited on previous jobs. The reported anecdotal trend was that personal protective equipment had become more common over the last 10 years but engineering control still remained limited.

## Discussion

This is the first case series that describes the re‐emergence of chronic silicosis within the tunnelling sector. As tunnelling is a major activity within large infrastructure and construction projects around Australia, this case series suggests that there may be a larger cohort of tunnellers with chronic silicosis who have yet to be diagnosed with silicosis due to their current asymptomatic states and the small numbers of persons trained to diagnose this condition, both clinically and radiologically [7]. With all participants presenting as clinically asymptomatic, the need for the rigorous screening workers in high‐risk occupations within the construction and infrastructure development sectors is highlighted by the case series.

We believe that an effective medical surveillance programme was able to be implemented in Queensland due to the increased awareness amongst employers, medical professionals, and radiologists who were qualified in ILO B reading. This increased awareness was a direct result of the cases of acute silicosis and coal workers pneumoconiosis which is unique to Queensland [3, 7, 8]. It is this improved medical surveillance which has allowed for the diagnosis of chronic silicosis.

Noting the limitations of a case series, possible hypotheses for the re‐emergence of chronic silicosis include the increased number of construction projects in areas with high silica content. Other hypotheses include poor occupational hygiene practices – such as suboptimal ventilation and over‐reliance on ineffective personal protective equipment. A further hypothesis could be that the published very low incidence rates resulted in a decreased perception of risk. This faulty perception could have led to a reduced focus and adherence on traditional occupational hygiene practices that have been demonstrated to be effective.

The medical surveillance programme that was undertaken looked at all workers who were employed at tunnelling sites. As such, the cohort from which our case series was drawn comprised of administrative personnel, senior and middle management, and ancillary site staff in addition to those persons who worked underground. We postulate that there may be a prevalence of up to 10% if the tunnellers were categorized into a homogenous exposure group.

Due to the specialized nature of tunnelling, tunnellers frequently move across various construction projects in Australia and are often employed by several different companies during their working career. Silicosis screening programmes are currently state‐based and therefore this cohort of workers are at this potential risk of being missed as they move across state boundaries. In addition, the state‐based nature of workers compensation schemes does not easily accommodate workers who have acquired silicosis across multiple jurisdictions.

This case series also identifies a need for improved dust monitoring programmes. This need is linked to not only preventing the causation of the occupational lung disease, but also in assisting fitness for work determinations and improving work‐related outcomes for these individuals. There will also need to be greater attention paid to engineering controls and personal protective equipment within these underground work environments.

In conclusion, the re‐identification of black lung and silicosis in the mining industry in Queensland in 2015 has led to a greater awareness of silicosis in other industries. In Queensland, screening for silicosis has been expanded beyond the coal industry to artificial stone workers, quarry workers, and hard rock miners. This cluster of eight cases in tunnellers supports the need for further expansion of screening in construction and infrastructure workers, particularly those in high‐risk roles such as those involved in underground tunnel projects. The re‐emergence of chronic silicosis will pose new and different healthcare challenges within this larger industry which is potentially fragmented in its regulation. It is envisaged that the increase in the chronic silicosis burden of disease will add to the growing public health interventions around pneumoconiosis over the coming years.

### Disclosure Statement

Appropriate written informed consent was obtained for publication of this case series and accompanying images.
